# Photocatalytic Cascade Reaction Driven by Directed Charge Transfer over *V*_S_‐Zn_0.5_Cd_0.5_S/GO for Controllable Benzyl Oxidation

**DOI:** 10.1002/advs.202207250

**Published:** 2023-05-01

**Authors:** Xue Bai, Mengyao She, Yali Ji, Zhe Zhang, Wenhua Xue, Enzhou Liu, Kerou Wan, Ping Liu, Shengyong Zhang, Jianli Li

**Affiliations:** ^1^ Chemistry Key Laboratory of Synthetic and Natural Functional Molecule of the Ministry of Education College of Chemistry & Materials Science Northwest University Xi'an 710127 P. R. China; ^2^ Key Laboratory of Resource Biology and Biotechnology in Western China Ministry of Education Biomedicine Key Laboratory of Shaanxi Province Lab of Tissue Engineering the College of Life Sciences Faculty of Life Science & Medicine Northwest University Xi'an 710069 P. R. China; ^3^ School of Chemical Engineering Northwest University Xi'an 710127 P. R. China; ^4^ Key Laboratory of Catalytic Materials and Technology of Shaanxi Province Kaili Catalyst & New Materials Co., Ltd. Xi'an 710201 P. R. China

**Keywords:** photocatalytic cascade reaction, proton‐coupled electron transfer, singlet O_2_, S vacancy

## Abstract

Photocatalysis is an important technique for synthetic transformations. However, little attention has been paid to light‐driven synergistic redox reactions for directed synthesis. Herein, the authors report tunable oxidation of benzyl to phenylcarbinol with the modest yield (47%) in 5 h via singlet oxygen (^1^O_2_) and proton‐coupled electron transfer (PCET) over the photocatalyst Zn_0.5_Cd_0.5_S (ZCS)/graphene oxide (GO) under exceptionally mild conditions. Theoretical calculations indicate that the presence of S vacancies on the surface of ZCS/GO photocatalyst is crucial for the adsorption and activation of O_2_, successively generating the superoxide radical (^•^O_2_^−^) and ^1^O_2_, attributing to the regulation of local electron density on the surface of ZCS/GO and photogenerated holes (h^+^). Meanwhile, accelerated transfer of photogenerated electrons (e^−^) to GO caused by the *π*–*π* stacking effect is conducive to the subsequent aldehyde hydrogenation to benzyl alcohol rather than non‐selective oxidation of aldehyde to carboxylic acid. Anisotropic charge transport driven by the built‐in electric field can further promote the separation of e^−^ and h^+^ for multistep reactions. Promisingly, one‐pot photocatalytic conversion of *p*‐xylene to 4‐methylbenzyl alcohol is beneficial for reducing the harmful effects of aromatics on human health. Furthermore, this study provides novel insights into the design of photocatalysts for cascade reactions.

## Graphical abstract

An approach in controllable benzyl oxidation is provided by photocatalytic synergy of ^1^O_2_ and proton‐coupled electron transfer in this work. Surface local electron density impressed by the regulation of S vacancy, as well as *π*–*π* interactions between GO and benzene ring facilitate the multistep reactions via separation of photogenerated e^−^ and h^+^.

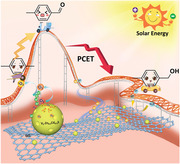

## 1 Introduction

**Scheme 1:**
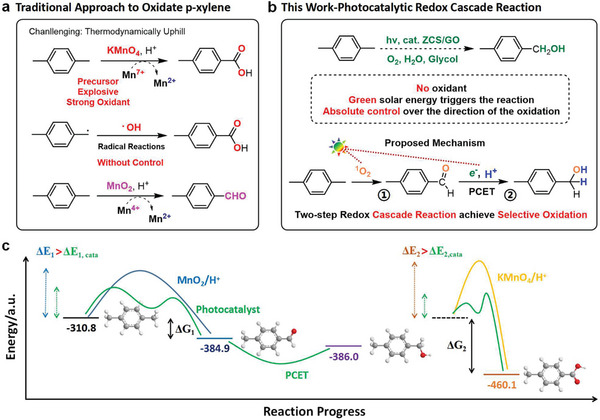
a,b) Comparison between traditional *p*‐xylene oxidation and the photocatalytic cascade reaction reported in this study and c) schematic of the trends of energy barriers during these two reactions.

Aromatic hydrocarbons pose significant threats to human health and the environment because of their carcinogenicities, among which *p*‐xylene is highly toxic because it damages the central nervous system and causes abnormal development of blood cells.^[^ ^1^, ^2^ ^]^ Traditional benzyl group oxidation involves several steps such as radical, nucleophilic, and elimination reactions, which require oxidants (KMnO_4_ and MnO_2_) and acidic conditions (**Scheme** **1**a). Nevertheless, a hidden safety hazard exists in the application of strong oxidants as KMnO_4_ is an explosive and precursor chemical. Additionally, radical reactions, in which the benzyl group is always oxidized to the carboxyl group without selectivity, are difficult to control. Moreover, the oxidation potentials of aldehydes are close to those of alcohols.^[^ ^3^, ^4^ ^]^ Therefore, optimizing traditional oxidation of *p*‐xylene and obtaining benzyl alcohol via an oxidation path are still considerable challenges.

Conversion of solar energy to chemical energy via artificial photosynthesis is a preferable approach to solve the energy consumption and pollutant discharge problems.^[^ ^5^, ^6^, ^7^, ^8^, ^9^, ^10^, ^11^ ^]^ Various reactive oxygen species (e.g., ^•^OH, ^•^O^−^_2_, and ^1^O_2_ free radicals) play important roles in photocatalytic oxidations.^[^ ^12^ ^]^ However, the oxidation capability of the majority of reactive oxygen species always exhibits nonselective complete mineralization, which may substantially decrease atom utilization. ^1^O_2_ acts as a mild yet efficient oxidant and is a versatile reactive oxygen species with applications in multiple organic transformations and photodynamic cancer therapy.^[^ ^4^, ^13^ ^]^ A recent study has verified that the *α*‐ethereal C—H bonds of aliphatic ethers can be selectively functionalized by ^1^O_2_ via one‐step direct insertion.^[^ ^14^ ^]^ Nevertheless, to date, this approach has not been applied to the oxidation of benzyl and toluene derivatives.

As the other half‐reaction, reduction simultaneously occurs during a photocatalytic reaction. In this regard, in situ hydrogenation in a photocatalytic system is expected to be a new way for solar energy utilization.^[^ ^15^, ^16^, ^17^ ^]^ To minimize the recombination of excited carriers, photocatalytic redox synergy is a promising approach for transforming and storing solar energy in chemical forms.^[^ ^18^, ^19^, ^20^ ^]^ Benzyl oxidation reported herein is performed under mild conditions using O_2_ as the O source, and controlled oxidation products are achieved owing to the synergistic effect of singlet oxygen (^1^O_2_) and proton‐coupled electron transfer (PCET, Scheme 1b). Thus, this photocatalytic synthesis route, relying on light as an energy source to drive reactions using water and O_2_ as atom sources, can be a viable “greener” alternative to traditional chemical methods. In contrast, photocatalysts can reduce the energy barrier of benzyl oxidation, leading to an exclusive benzyl alcohol product (i.e., 4‐methylbenzyl alcohol) with appropriate yield under mild reaction conditions (Scheme 1c); the storage of 4‐methylbenzyl alcohol is convenient than that of flammable *p*‐xylene; thus, the proposed strategy can afford new functionalities for industrial applications.

In this study, we aim to understand the surface and interface characteristics, including the interaction of photocatalysts with O_2_ and behaviors of the carriers in the heterojunction of photocatalysts, which significantly determine the type of species involved and activities of reactive oxygen free radicals in the corresponding oxidation. Herein, we regulated the concentrations of S vacancies (*V*_S_) on the surface of Zn_0.5_Cd_0.5_S (ZCS)/*x*‐graphene oxide (GO) (where *x* represents the GO:ZCS mass ratio) used as an efficient photocatalyst to prevent excessive oxidation of the benzyl group. Particularly, a crucial intermediate of ^1^O_2_ was observed, and PCET was conducive to the subsequent hydrogenation of aldehyde to benzyl alcohol rather than the carboxylation of aldehyde. Synergistic control of both heterojunction structures and surface electrons provided by *V*_S_ considerably enhanced the cascade reaction activity of ZCS/*x*‐GO. Fortunately, this study provides novel insights into the design of photocatalysts for cascade reactions and a new strategy for the controllable oxidation of *p*‐xylene, which can serve as a promising alternative to the traditional protocol of petroleum derivative synthesis.

## 2 Results and Discussion

### 2.1 Dual Role of GO in Structure Defect Restoration and *V*_S_ Production in ZCS/*x*‐GO

**Figure 1:**
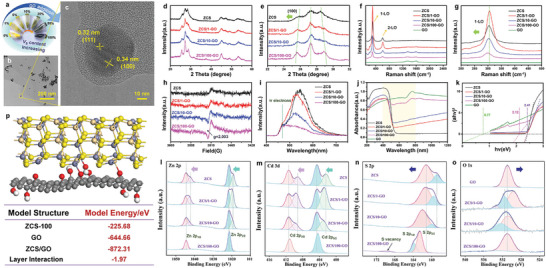
a) Appearance colors of ZCS/*x*‐GO. b) TEM images, c) HRTEM images, d) XRD patterns, e) partially magnified XRD patterns, f) Raman spectra, and g) partially magnified Raman spectra of ZCS/*x*‐GO. h) EPR, i) PL, j) UV‐vis absorption spectra, and k) (*αhν*)^2^ versus (*hν*) plots of hexagonal ZCS and ZCS/*x*‐GO. High‐resolution XPS spectra of l) Zn 2p, m) Cd 3d, n) S 2p, and o) O 1s for ZCS/*x*‐GO heterojunctions. p) Theoretically simulated structure and calculation of the energy of interlayer interaction between ZCS and GO.

ZCS/*x*‐GO heterojunction samples with different GO:ZCS mass ratios were prepared by regulating the amount of GO (**Figure** **1**a). To confirm the amalgamation of Zn, Cd, and S to form ZCS nanoparticles, scanning electron microscopy (SEM) and transmission electron microscopy (TEM) images with corresponding metal mappings and energy spectra were acquired (Figure S1a–e, Supporting Information). Furthermore, the TEM (Figure 1b) image exhibited 2D film morphology of GO and 0D nanoparticle morphology of ZCS. N_2_ adsorption/desorption isotherms and Barrett–Joyner–Halenda pore size distribution maps indicated micropore structures of ZCS/*x*‐GO with average pore widths of 1.4916 nm (Figure S1f,g, Supporting Information). Clear lattice fringes with spacings (*d*) of 0.32 and 0.34 nm, corresponding to the (111) and (100) planes of hexagonal ZCS, were observed in the high‐resolution TEM (HRTEM) image (Figure 1c).^[^ ^21^, ^22^ ^]^ Selected area electron diffraction results implied that the polycrystalline structure of ZCS improved upon the addition of GO (Figure S2, Supporting Information), which was verified by their diffraction patterns (the diffraction rings demonstrated polycrystalline natures of ZCS/*x*‐GO) and corresponding radial intensity profiles. X‐ray diffraction (XRD) patterns and Raman spectra of the samples were consistent with those of the hexagonal Cd_0.5_Zn_0.5_S solid (Figure 1d–f).^[^ ^23^, ^24^, ^25^ ^]^ The peak related to the (100) lattice plane and 1‐LO peak continuously shifted to lower degrees and frequencies, respectively, with an increase in the GO content (Figure 1e–g) due to interaction between ZCS and GO. Moreover, high‐angle shifts of the XRD peaks indicated a better interlayer between ZCS and GO (Figure 1e), which was consistent with the HRTEM results. Electron paramagnetic resonance (EPR) spectroscopy was performed to verify the existence of *V*_S_, and a signal at *g* = 2.003 with different intensities was detected for ZCS/*x*‐GO (Figure 1h), implying that the concentrations of *V*_S_ in ZCS increased upon the introduction of GO.^[^ ^26^, ^27^ ^]^ The competition between the improvement of charge separation by the photoactivated carriers trapped by vacancies and transformation of these carriers into recombination centers determines the overall effects of photocatalytic reactions. Figure 1h depicts the steady‐state photoluminescence (PL) spectra of ZCS/*x*‐GO, which exhibit peaks centered at 520 nm with the intensity order of ZCS > ZCS/1‐GO > ZCS/10‐GO > ZCS/100‐GO. The broad emissions were assigned to surface defects.^[^ ^25b^ ^]^ More importantly, the intense emission peak at 469 nm was related to the *π* electronic transition in GO.^[^ ^28^ ^]^ These results suggested a more effective separation of photogenerated electron (e^−^)–hole (h^+^) pairs owing to the existence of *V*_S_ and ZCS/*x*‐GO heterojunctions in ZCS/*x*‐GO. Furthermore, the addition of a sufficient amount of GO enhanced the visible light absorption ability (yellow area in Figure 1j) and narrowed the bandgap of ZCS (from 2.41 to 2.12 eV), which was estimated using the Kubelka–Munk function: (*αhν*)*^n^* = *A*(*hν – E*_g_) (Figure 1k).

Figure 1l–o shows the high‐resolution X‐ray photoelectron spectra (XPS) of Zn 2p, Cd 3d, S 2p, and O 1s of ZCS/*x*‐GO, respectively. Generally, the peaks located at 1021.5 and 1044.4 eV were ascribed to Zn 2p3/2 and Zn 2p1/2 (Figure 1l), respectively, and the peaks observed at 404.6 and 411.3 eV corresponded to Cd 3d5/2 and Cd 3d3/2 (Figure 1m). Moreover, after the incorporation of GO into ZCS, the peaks located at 1019.2 and 1042.2 eV shifted to lower binding energies owing to the increase in electron density around the Zn atoms caused by surface defects. Additionally, the same effect was noticed for the Cd atoms (402.4 and 409.2 eV). Furthermore, these peaks shifted to high binding energies with an increase in the amount of GO, which might be attributed to the higher electronegativity of O (3.44) than those of Zn (1.65), Cd (1.69), and S (2.58); thus, the introduction of O into ZCS might decrease the electron densities of Zn, Cd, and S atoms. The energy of interlayer interaction between ZCS and GO was determined to be −1.97 eV via theoretical calculations (Figure 1p); therefore, a stable combination of ZCS and GO with covalent linkages was predicted. Additionally, the peak related to the S atom shifted to higher binding energy (Figure 1n), whereas that of the O atom shifted to lower binding energy (Figure 1o). Note that ZCS/100‐GO possesses a clean surface structure, and the peak at 169.7 eV corresponds to *V*_S_. Therefore, in the case of *V*_S_‐ZCS/100‐GO with an appropriate amount of GO, the structural defects of ZCS were provided, and the concentration of *V*_S_ was improved as well.

### 2.2 Regulation of Surface Local Electron Density by *V*_S_ for Promoting O_2_ Activation

**Figure 2:**
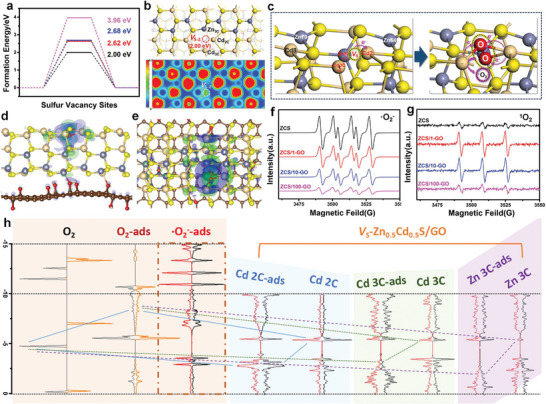
a) Simulated formation energies of *V*_S_. b) Structure of the *V*_S_‐ZCS/GO surface with the corresponding charge density contour pattern. Electron flow diagram for the c) introduction of *V*_S_ and adsorption of O_2_. Charge density difference and O_2_ PDOS of the *V*_S_‐ZCS (100) surface with d) side and e) top views, where green and blue areas represent charge accumulation and depletion in the space, respectively. f,g) EPR spectra of ^•^O_2_^−^ and ^1^O_2_ for the as‐prepared samples, respectively. h) DOS and adsorption activity analysis at the molecular level.

O_2_ acts as a terminal oxidant for catalyst reoxidation and affords an active superoxide radical anion.^[^ ^29^, ^30^ ^]^ Vacancies are intrinsic defects present in crystals; they not only enhance charge transfer, inhibiting the recombination of e^−^ and h^+^, but also play vital roles as adsorption sites, thereby increasing the reactivities.^[^ ^31^, ^32^ ^]^ When *V*_S_ are present, the absence of anions may endow *V*_S_ with affinities toward electrons, i.e., *V*_S_ serve as active sites for electron utilization.^[^ ^33^ ^]^ To a certain extent, the electron densities of ZCS/*x*‐GO reflect the active sites on the surface area. The abovementioned analysis and density functional theory (DFT) calculation data (**Table** **1**) indicated excess electron density of Cd (11.69) around *V*_S_ when compared with that in the case of ZCS (11.16), attributed to the construction of *V*_S_ in ZCS/*x*‐GO. In fact, the use of *V*_S_ to adsorb and activate O_2_ has already been reported, and the resulting systems have been highly effective for selective photooxidations.^[^ ^34^ ^]^ However, to date, the relationship between the types of activated free radicals and local electron distribution on the photocatalyst surface has not been investigated. In this study, we have demonstrated that the surrounding disorder surface localized states provide the driving force for the reduction of O_2_ (Table 1 and **Figure** **2**c).

**Table 1:** Comparison of the Bader charges at different adsorption sites

Sites samples	Cd2	Cd8	Zn64	Zn70	C92	C101	Cd3C‐2nd	Zn3C‐2nd
ZCS/GO	11.16	11.16	11.16	11.16	3.70	3.70	∖	∖
*V*_S_‐ZCS/GO	**11.69**	11.17	11.17	11.16	3.75	3.73	**11.31**	**11.22**
*V*_S_‐O_2_	**10.97**	11.16	11.16	11.16	3.73	3.61	**11.04**	**11.07**
*V*_S_‐OOH	**11.00**	11.16	**11.11**	**11.12**	3.66	3.74	**11.07**	**11.04**

Obvious changes of Bader charges at different adsorption sites are highlighted in bold.

Theoretical calculations simulated four different atomic configurations of the *V*_S_‐ZCS/(100)‐GO surface according to the *V*_S_ sites (Figure S3, Supporting Information), where *V*_S‐2_‐ZCS/(100)‐GO was preferentially introduced due to its lowest formation energy (2.0 eV) (Figure 2a). Electron localization function analysis implied that the electronic density of *V*_S‐2_ was lower than those of the surrounding atoms, and reducibility spread around the metal atoms, such as Cd_2c_ and Zn_3c_, as marked in Figure 2b. To further understand the structure–property relationship in ZCS/GO using *V*_S‐2_, the Bader charges of Cd, Zn, and C atoms at different adsorption sites of ZCS/GO and *V*_S_‐ZCS/GO for O_2_ molecules are presented in Tables S1 and S2, Supporting Information. The Bader charge on the Cd_2C_ active site was higher owing to the presence of *V*_S_ as compared to that on the Cd_3C_ active site (without *V*_S_). Consequently, the metal sites around *V*_S_ were partially reduced, and Cd_2C_ was substantially reduced; in contrast, new sites (Cd_3C_ and Zn_3C_) emerged at the second layer (Table 1). More importantly, the interaction of O_2_ with *V*_S_ resulted in charge redistributions, which demonstrated the potentials of *V*_S_ as active sites for the production of superoxide radicals (Figure 2c), and charge density difference calculations were adopted to trace the interfacial charge transfer and reveal the significantly promoted thermodynamics of ^•^O_2_^−^ over the surface of *V*_S_‐ZCS/GO (Figure 2d,e). After the adsorption of O_2_, instant charge back‐donation from Cd and Zn atoms around *V*_S_ to O_2_ occurred, which was indicated by the localized electron depletion on Cd_2C_ and Zn_3C_ and accumulation of electrons on the coordinating O_2_. Therefore, *V*_S‐2_ in *V*_S‐2_‐ZCS/GO offers a suitable adsorption site for O_2_ molecules and easily transforms O_2_ to ^•^O_2_^−^ because the local electronic structure is favorable for electron transfer.

Accordingly, electron spin resonance measurements were conducted to confirm the generation of ^•^O_2_^−^, ^1^O_2_ (Figure 2f,g), and ^•^OH (Figure S4, Supporting Information), clarifying that the active oxygen species in our system were ^•^O_2_^−^ and ^1^O_2_. Comparison of the signal strengths of ^•^O_2_^−^ and ^1^O_2_ implied that pure ZCS produced the highest number of ^•^O_2_^−^ and lowest number of ^1^O_2_ because of the relatively large amount of e^−^. Clearly, the ^•^O_2_^−^ accumulative intensity decreased with an increase in the amount of GO owing to the rapid separation of e^−^ and h^+^. Nevertheless, because of shielding effect of the large amount of GO, the capacity of *V*_S_‐ZCS/100‐GO to generate ^•^O_2_^−^ and ^1^O_2_ decreased. Calculations of the adsorption energies of O_2_ molecules and desorption energies of ^•^O_2_^−^ on *V*_S_‐ZCS/GO suggest that the strong adsorption of O_2_ leads to preferential formation of ^•^O_2_^−^ (Table S3, Supporting Information). Furthermore, high desorption energies of ^•^O_2_^−^ raise its concentrations, which provides an advantage in further oxidation to ^1^O_2_ by photogenerated h^+^ on the surface of ZCS.^[^ ^35^ ^]^ These findings are consistent with those of the EPR measurements reported in previous studies. Moreover, the adsorption states of O_2_ over (*V*_S_)‐ZCS/GO at different sites were systematically simulated (Figures S5–S10, Supporting Information), and the schematic of the filling of the lowest unoccupied molecular orbital by lone pair electrons further verified that the regulation of the surface local electron density triggered by *V*_S_ promoted O_2_ reduction (Figure 2h). Our proposed mechanism involves the conversion of O_2_ to ^•^O_2_^−^ and sequential generation of ^1^O_2_^[^ ^36^ ^]^ on ZCS/100‐GO under light irradiation (Figure 4d), which was supported by the calculated partial density of states (PDOS) at different sites on *V*_s_‐ZCS/GO for the s, p, and d orbitals (Figure S11, Supporting Information). Additionally, the local DOS at the Fermi level decreased upon the introduction of *V*_s_.

### 2.3 Promotion of C=O Hydrogenation by the Acceleration of e^−^ Transfer and *π*–*π* Stacking Effect

**Figure 3:**
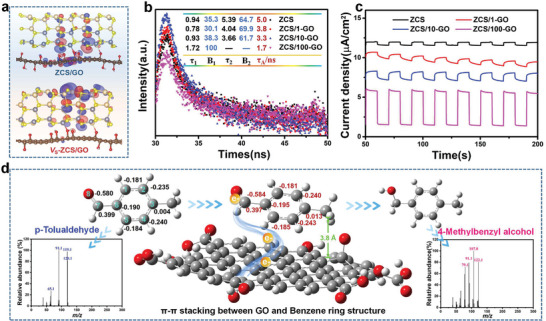
a) Simulated structures and corresponding diagram model crystal orbit phase images of ZCS/GO and *V*_s_‐ZCS/GO heterojunctions. b) Time‐resolved transient PL decay and c) transient photocurrent spectra of the prepared samples. d) Simulated structures of the synthesized samples for *π*–*π* stacking and electron transfer pathway, and mass spectra of photocatalytic products.

Highest occupied crystal orbital and lowest unoccupied crystal orbital energy levels of *V*_S‐2_‐ZCS/GO were better separated than those of ZCS/GO (**Figure** **3**a); this suggested that the doping of *V*_S‐2_ enhanced the local electrostatic potential (Figure S12, Supporting Information), and the e^−^–h^+^ pairs are superior in spatially separating. To further estimate the behaviors of photoexcitons, time‐resolved transient fluorescence (TRPL) and average PL lifetime (*τ*_A_) were measured. *τ*_A_ values of ZCS, ZCS/1‐GO, and ZCS/10‐GO were evaluated according to the equation $\tau_{A}\;=\;\frac{B_{1}\cdot \tau_{1}^{2}\;+\;B_{2}\cdot \tau_{2}^{2}}{B_{1}\cdot \tau_{1}\;+\;B_{2}\cdot \tau_{2}}$, whereas *τ*_A_ of ZCS/100‐GO was determined using the first‐order formula $\tau_{A}\;=\;\frac{B_{1}\cdot \tau_{1}^{2}}{B_{1}\cdot \tau_{1}}$ because of the minimal interband recombination of the excited state carriers in ZCS/100‐GO. As shown in the inset table of Figure 3b, the shortest of carriers in ZCS/100‐GO (1.7 ns) confirmed the rapid transfer of photogenerated electrons transfer to GO,^[^ ^37^, ^38^ ^]^ which thereby suppressed the recombination of e^−^ and h^+^ and implied the most efficient electron transfer from ZCS to GO in a nonradiative quenching pathway. This result is in adequate accordance with the transient photocurrent spectra depicted in Figure 3c, which reveals the promoted production and transfer of photogenerated electron–hole pairs in the heterostructures.^[^ ^39^ ^]^ The binding of GO to arenes mostly occurs via weak noncovalent interactions including *π*–*π* stacking, and this interaction has the ability to facilitate the fast electron transport.^[^ ^40^ ^]^ The simulated structures of benzaldehyde, 4‐methyl‐ before and after the approach to GO verified the changes in the electronegativities of C and O atoms (marked as numbers 1–8 in Figure 3d), which enabled the transfer of electrons from GO to benzaldehyde, 4‐methyl‐. Furthermore, the distance between benzaldehyde, 4‐methyl‐ and GO was close to that of *π*–*π* stacking (3.8 Å). Mass spectra of benzaldehyde, 4‐methyl‐ and 4‐methylbenzyl alcohol are depicted in the inset of Figure 3d, which were obtained by gas chromatography of the photocatalytic product mixture (Figure S13, Supporting Information) with retention times of 10.19 and 11.92 min, respectively.

**Figure 4:**
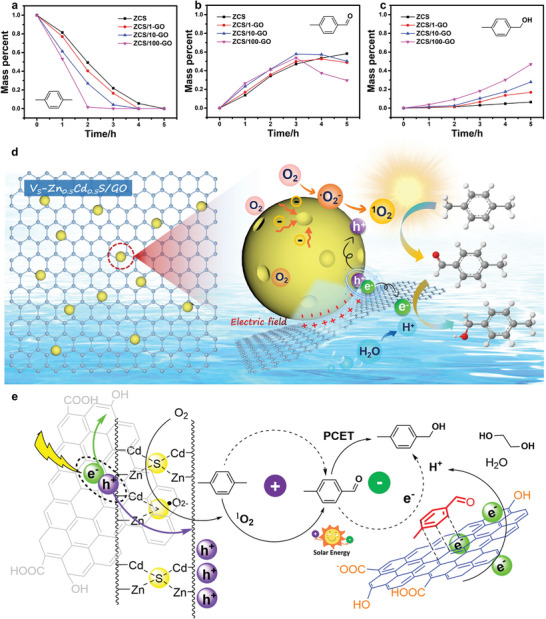
a–c) Analysis of the reaction dynamics of *p*‐xylene, benzaldehyde, 4‐methyl‐ and 4‐methylbenzyl. d,e) Proposed photocatalytic cascade reaction over *V*_S_‐ZCS/GO for the controllable oxidation in pure water.

Particularly, our calculation results were in excellent agreement with the experimental results. In photocatalysis, rapid charge transfer and long lifetimes of the intermediate species are required for redox reactions. Accordingly, the GO nanosheets provided sufficient e^−^ and H^+^ evolution active sites for PCET (**Figure** **4**d and Figure S14, Supporting Information). In addition to facilitating charge separation, PCET can efficiently promote the activity of ZCS/GO for 4‐methylbenzyl alcohol formation, and analysis of the corresponding mechanism is described hereinafter.

### 2.4 Synergistic Effect of Photocatalytic Redox in the Overall System

Based on the abovementioned information, we performed comprehensive investigations to understand the mechanism of the synthesis of 4‐methylbenzyl alcohol. The addition of the radical scavengers 5,5‐dimethyl‐1‐pyrroline *N*‐oxide and 4‐hydroxy‐2,2,6,6‐tetramethylpiperidine to the reaction system significantly suppressed the formation of benzaldehyde, 4‐methyl‐, which proved that ^1^O_2_ was involved in the oxidation of the benzyl group to the aldehyde group. The introduction of an electron scavenger (nitrobenzene) into the system containing either ZCS or *V*_S_‐ZCS/GO inhibited the formation of H_2_ and considerably decreased the production rate of 4‐methylbenzyl alcohol; nevertheless, it slightly influenced the formation of benzaldehyde, 4‐methyl. Therefore, as the key chemical reagents for hydrogenation, e^−^ produced over GO and H^+^ in water are reasonably involved in the synthesis of 4‐methylbenzyl alcohol. The above‐mentioned results suggested that the formation of benzaldehyde, 4‐methyl‐ and 4‐methylbenzyl alcohol proceeded via radical intermediates and electrons, respectively. Thus, the electronic structure tailored by *V*_S_ introduction, as indicated earlier, substantially affects photocatalytic synergistic redox reactions over ZCS/GO (Figure 4e). In the presence of O_2_, ^1^O_2_ is considerably produced along with the enrichment of ^•^O_2_^−^ on the catalyst surface. Furthermore, the *π*–*π* stacking effect accelerates e^−^ separation, facilitating hydrogenation. The integration with photocatalytic PCET in aqueous media is speculated to extend the chemical reaction to two steps: oxidation of the benzyl group to the aldehyde group and hydrogenation of the aldehyde group to benzyl alcohol.

To gain further insight into the reaction mode, 5 mg catalyst was dispersed into a 20 mL H_2_O solution comprising 0.5 mL ethylene glycol and 0.5 mL *p*‐xylene, and the mixture was irradiated with a Xe lamp for 5 h under atmospheric temperature and normal pressure conditions. The products obtained per hour were analyzed by gas chromatography–mass spectrometry (GC–MS) using a quadrupole mass spectrometer detector. Oxidation of the benzyl to aldehyde occurred in the first 3 h over all catalysts (Figure 4a–c). Thereafter, the yield of aldehyde gradually decreased with an increase in reaction time over ZCS/1‐GO, ZCS/10‐GO, and ZCS/100‐GO, and ZCS/100‐GO demonstrated the clearest inflection point when compared with those of the other catalysts. Notably, the yield of 4‐methylbenzyl alcohol increased along with a decrease in the amount of benzaldehyde, 4‐methyl‐, which almost reached 47% in 5 h over ZCS/100‐GO.

An overall photocatalytic process involves reduction and oxidation, where *p*‐xylene is oxidated to obtain 4‐methylbenzyl alcohol (Figure 4d), due to photogenerated anisotropic charge transport driven by the built‐in electric field over the ZCS/GO interface, which promoted the separation of e^−^ and h^+^ for these multistep reactions. Therefore, subsequently, the aldehyde is converted to benzyl alcohol via hydrogenation rather than to carboxylic group via nonselective oxidation. Specifically, under comparatively ideal charge generation, further separation of e^−^ and h^+^ and their consumption at the multiphase interface determine the photocatalytic efficiency of the catalyst (Figure 4e). According to this perspective, the cascade reaction via ^1^O_2_ and PCET and facilitated charge separation can efficiently enhance the photocatalytic activity of the catalyst for 4‐methylbenzyl alcohol formation. Additionally, a photocatalytic cascade reaction for the controllable oxidation of *p*‐xylene in pure water is reported for the first time in this study.

## 3 Conclusion

In summary, herein, we report a unique photocatalytic behavior of *V*_S_‐ZCS/GO via the successful application of manipulated benzyl group oxidation by a cascade reaction via ^1^O_2_ and PCET. Photoinduced charge separation occurring over *V*_S_‐ZCS/GO hybrid simultaneously creates reduction and oxidation centers. Moreover, *π*–*π* interactions between GO and the benzene ring removes the spatial restriction, thereby facilitating the attack of interfacial charges and enhancing the photocatalytic hydrogenation activity of *V*_S_‐ZCS/GO. Accordingly, a one‐pot two‐step reaction strategy over *V*_S_‐ZCS/GO was proposed to successfully transform a benzyl group into an aldehyde group and the aldehyde group to benzyl alcohol via oxidation and hydrogenation, respectively. This photocatalytic synergistic synthesis offers a new strategy for the controllable oxidation of *p*‐xylene.

## 4 Experimental Section

### Sample Preparation

Typically, 1 mmol CdCl_2_⋅2.5H_2_O and 1 mmol Zn(Ac_2_)⋅2H_2_O were dispersed in 50 mL deionized (DI) water followed by stirring for 5 min. Subsequently, 4.5 mL NaOH (4.5 mmol) solution was introduced dropwise into the resulting suspension, and then, the resulting mixture was transferred to a 100 mL stainless steel autoclave. The hydrothermal reaction system was maintained at 180 °C for 24 h. Finally, the obtained precipitates were acquired by centrifugation and washed more than three times with water. ZCS powder was achieved as the final product and dried at 60 °C for 6 h.

GO was fabricated by ultrasonic stirring of graphite oxide (Aladdin Biochemical Technology Co., LTD., 7782‐42‐5) and mixed with a certain amount of as‐prepared ZCS in DI water followed by stirring for 30 min. The resulting homogenous suspension was transferred to a stainless steel autoclave with a capacity of 70 mL. Subsequently, the autoclave was sealed and maintained at 180 °C for 12 h followed by cooling to room temperature. Finally, the resulting samples were obtained by centrifugation, washed several times with DI water, and dried in a drying oven.

### Characterization

Crystalline phases, morphologies, and textural characteristics of the acquired samples were examined by powder XRD (Smart Lab SE, Rigaku), TEM (Tecnai G2 F30), and SEM (Carl Zeiss Sigma) combined with energy dispersive spectroscopy. Specific surface areas of the samples were determined by N_2_ adsorption/desorption (Quantachrome NOVA 2000e). Contact angles were measured in soil using a contact angle meter (JY‐82B Kruss DSA). XPS (Kratos AXIS NOVA spectrometer) was conducted to examine the chemical compositions of products. UV‐vis spectrophotometry (Shimadzu UV‐3600) was performed to analyze the photo‐response properties of catalysts. The PL spectra were obtained using Hitachi F‐7000. TRPL spectra were acquired using a spectrofluorometer (FLS920, Edinburgh). EPR spectroscopy (Bruker ELEXSYS‐II E500) was conducted to investigate the active free radicals.

### Photocatalytic Measurements

Photocatalytic measurements were performed using an online detection system (Lab solar III‐AG, Beijing Perfect Light Technology Co. Ltd., China) connected to a gas chromatograph (Techcomp, GC7900). Herein, 5 mg ZCS/GO photocatalyst, 0.5 mL ethanediol, 0.5 mL *p*‐xylene, and 20 mL H_2_O were mixed for reactions. A 300 W Xe lamp (wavelength: 350–780 nm) was used as a light source and placed 15 cm away from the liquid level. The reaction temperature was controlled below 40 °C by cycling water. Products in the liquid phase were achieved hourly and identified by GC–MS (Agilent 7890A GC–5975C MS).

### Calculation Method

First‐principles calculations were conducted to determine the formation energies of the radicals. The projector augmented wave approach^[^ ^41^ ^]^ implemented in the Vienna ab initio package (VASP)^[^ ^42^ ^]^ was used to treat the valance electrons described by cut‐off plane waves and the core electrons expressed by pseudo wavefunctions. The Perdew–Burke–Ernzerhof functional based on the generalized gradient approximation was employed to calculate the exchange‐correlation interaction.^[^ ^43^ ^]^ The plane wave basis set was limited by an imposed cut‐off energy (*E*_cut_) of 400 eV. The 3 × 1 × 1 K points in the Γ‐centered scheme in the Brillouin zone were chosen for structural optimization, and the energy between two consecutive self‐consistent steps was less than 10^−6^ eV. The sole Γ point was selected for structural optimization, and the self‐consistent force was less than 0.05 eV Å^−1^. The energy between two consecutive self‐consistent steps was less than 10^−5^ eV. The DFT + U method with the on‐site Coulomb correction *U*_eff_ was utilized for the Zn 3d and Cd 4d electrons. *U*_eff_ values were 6 and 2.1 eV for Zn 3d and Cd 4d electrons according to previous theoretical investigations.^[^ ^44^, ^45^ ^]^ Grimme's zero‐damping DFT‐D3 method was used to calculate the van der Waals corrections.^[^ ^46^, ^47^ ^]^

## Conflict of Interest

The authors declare no conflict of interest.

## Supporting information

Supporting Information

## Data Availability

Research data are not shared.
