# Supplementary material for: Photocatalytic Cascade Reaction Driven by Directed Charge Transfer over V S‐Zn0.5Cd0.5S/GO for Controllable Benzyl Oxidation
Source: Adv Sci (Weinh). 2023 May 1;10(20):2207250. doi: 10.1002/advs.202207250 (PMC10369240; doi:10.1002/advs.202207250)

ADVANCED SCIENCE
Open Access

Supporting Information

for *Adv. Sci.*, DOI 10.1002/advs.202207250

Photocatalytic Cascade Reaction Driven by Directed Charge Transfer over V_S-$Zn_{0.5}Cd_{0.5}S$/GO for Controllable Benzyl Oxidation

Xue Bai, Mengyao She, Yali Ji, Zhe Zhang, Wenhua Xue, Enzhou Liu, Kerou Wan, Ping Liu*, Shengyong Zhang and Jianli Li**

Supporting Information

Photocatalytic Cascade Reaction Driven by Directed Charge Transfer over V_S-$Zn_{0.5}Cd_{0.5}S$ /GO for Controllable Benzyl Oxidation

Xue Bai, Mengyao She, Yali Ji, Zhe Zhang, Wenhua Xue, Enzhou Liu, Kerou Wan, Ping Liu*, Shengyong Zhang, and Jianli Li**

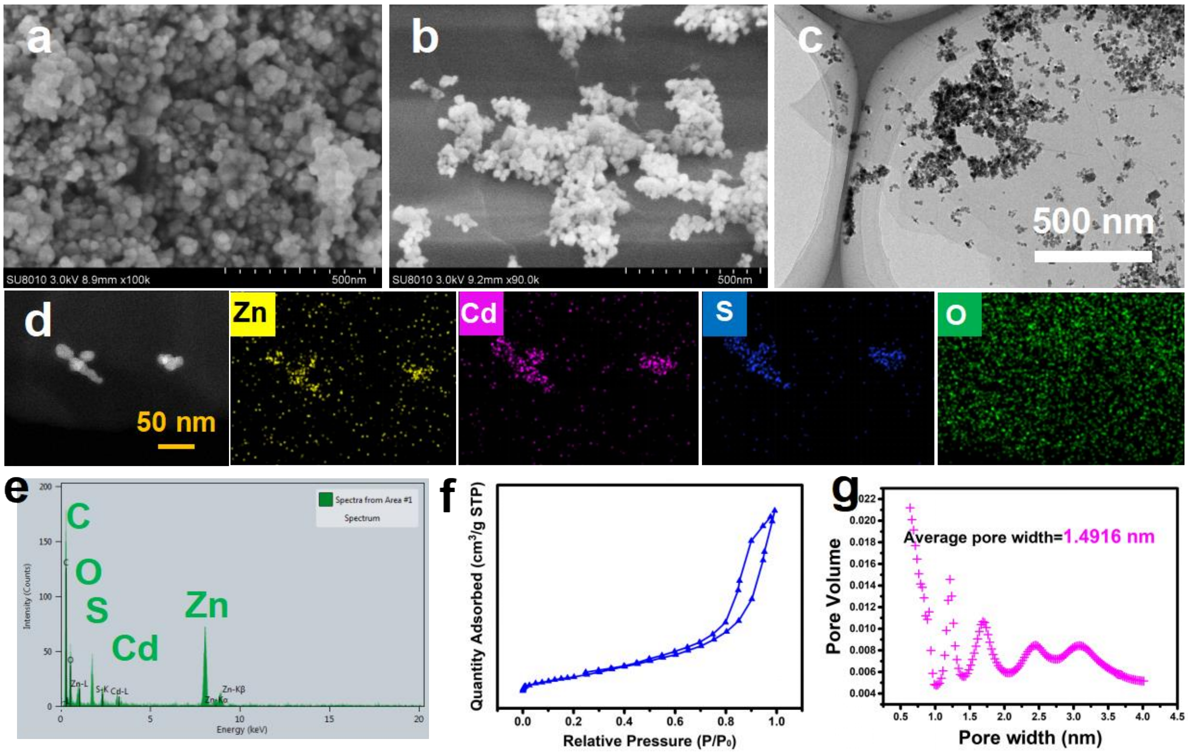

Figure S1. a-b) SEM, c) TEM images with corresponding mental mapping d) and e) energy spectra of Zn, Cd, and S elements, f) N_2 adsorption-desorption isotherms and g) Barrett-Joyner-Halenda pore size distribution maps.

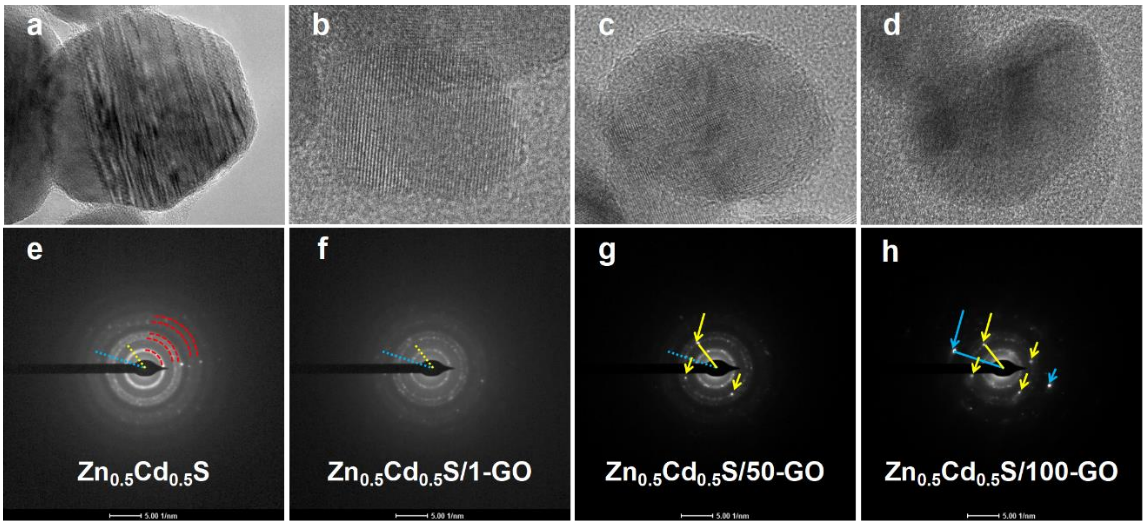

Figure S2. Electron diffraction (SAED) of different samples.

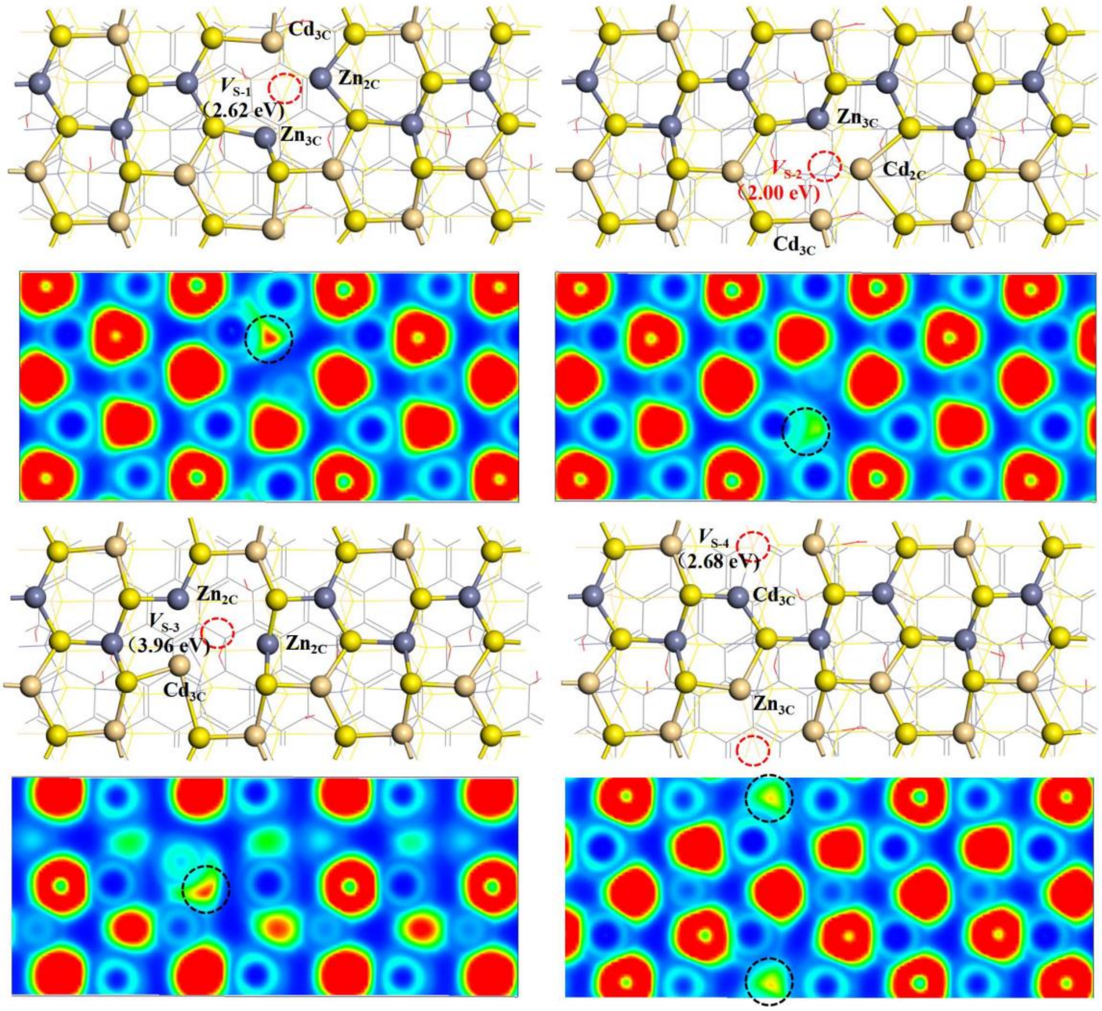

Figure S3. Electron localization function (ELF) analysis of V_S-$Zn_{0.5}Cd_{0.5}S$-(100)/GO surface according to the V_S sites.

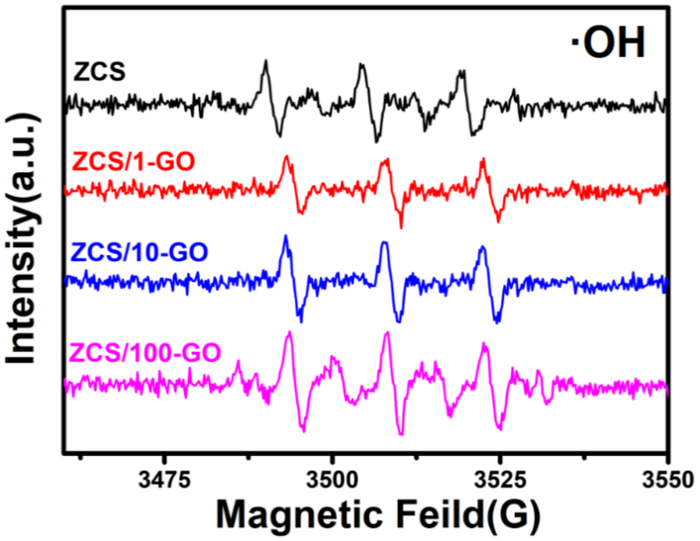

Figure S4. ESR measurement was employed to confirm the generation of ·OH.

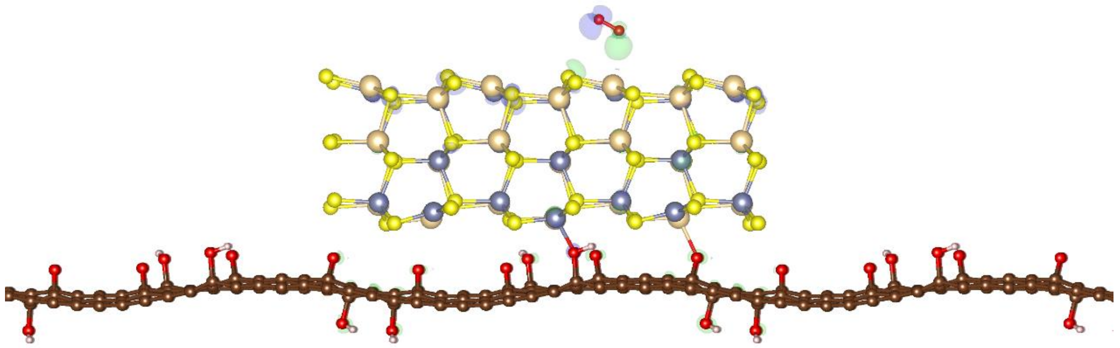

Figure S5. Theoretical simulation of $Zn_{0.5}Cd_{0.5}S$-(100)/GO-Cd-O_2.

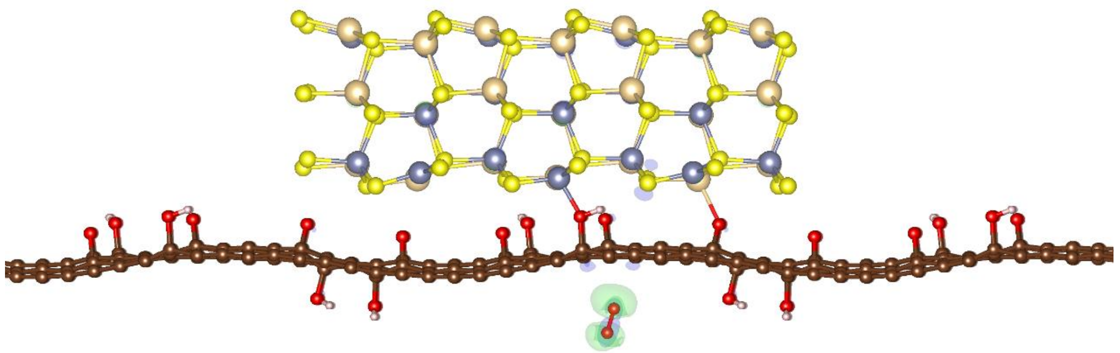

Figure S6. Theoretical simulation of $Zn_{0.5}Cd_{0.5}S$-(100)/GO-C-O_2.

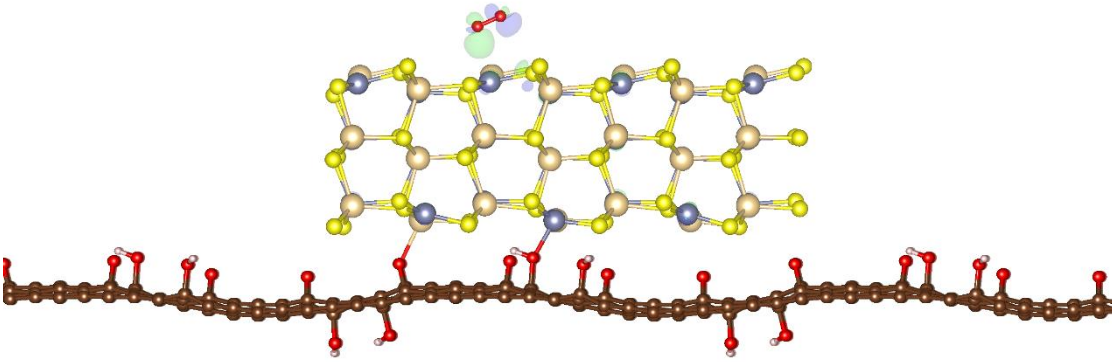

Figure S7. Theoretical simulation of $Zn_{0.5}Cd_{0.5}S$-(100)/GO-Zn-O_2.

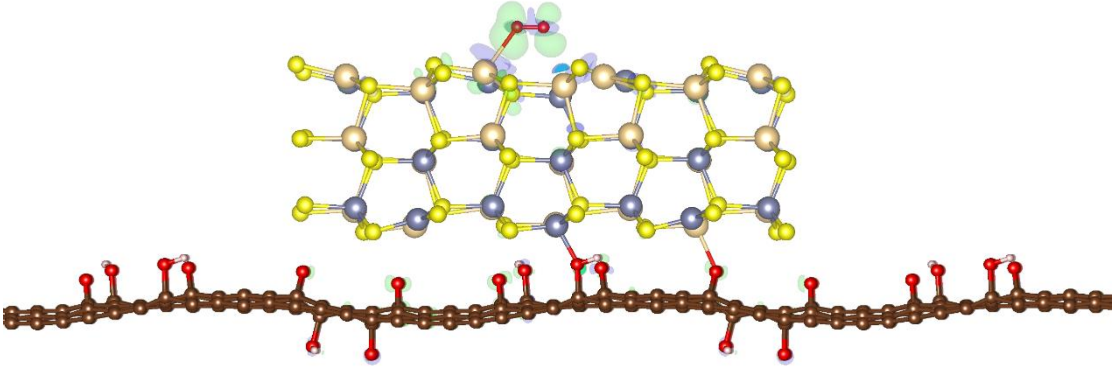

Figure S8. Theoretical simulation of V_S-$Zn_{0.5}Cd_{0.5}S$-(100)/GO-Cd3C-O_2.

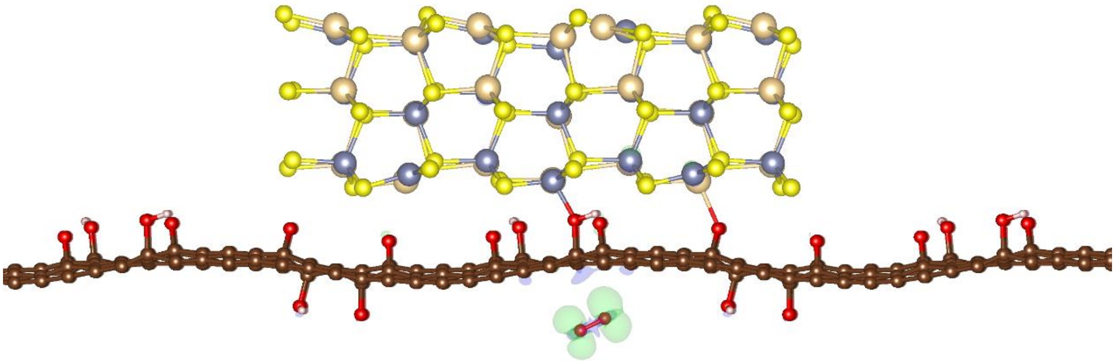

Figure S9. Theoretical simulation of V_S-$Zn_{0.5}Cd_{0.5}S$-(100)/GO-C-O_2.

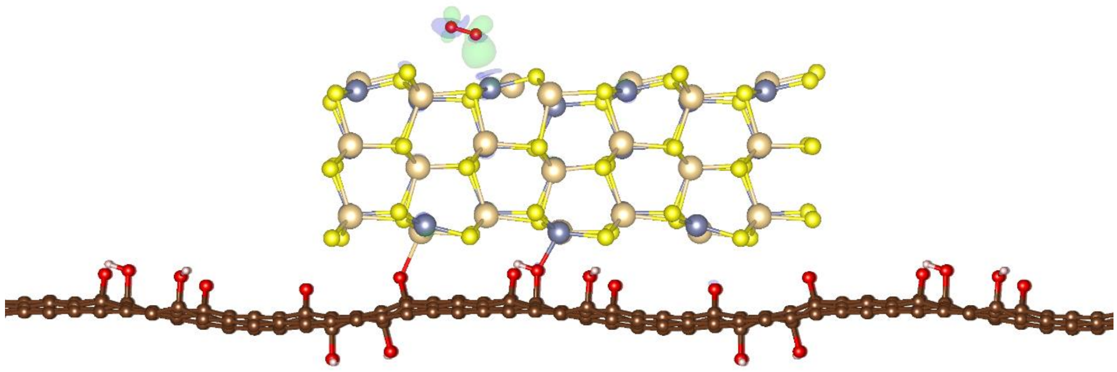

Figure S10. Theoretical simulation of V_S-$Zn_{0.5}Cd_{0.5}S$-(100)/GO-Zn-O_2.

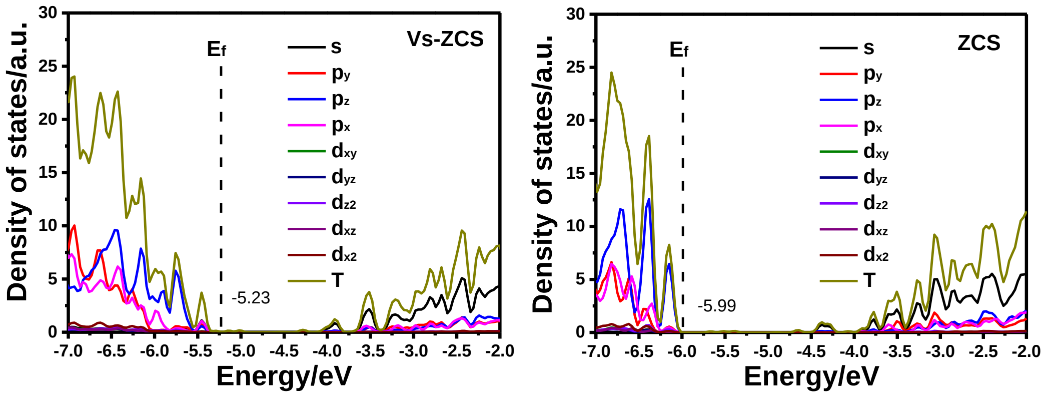

Figure S11. The partial density of states (PDOS) of different dislocations in V_s-ZCS/GO and ZCS/GO for the s, p, d orbitals.

Partial density of states (PDOS) of O_2 contained two occupied spin-up majority states and one occupied spin-down minority state below the Fermi energy, as well as an empty spin-down minority states near the conduction band, revealing the filling of one O_2 $2\pi^*$ orbital toward the $\bullet O_2^-$ formation.

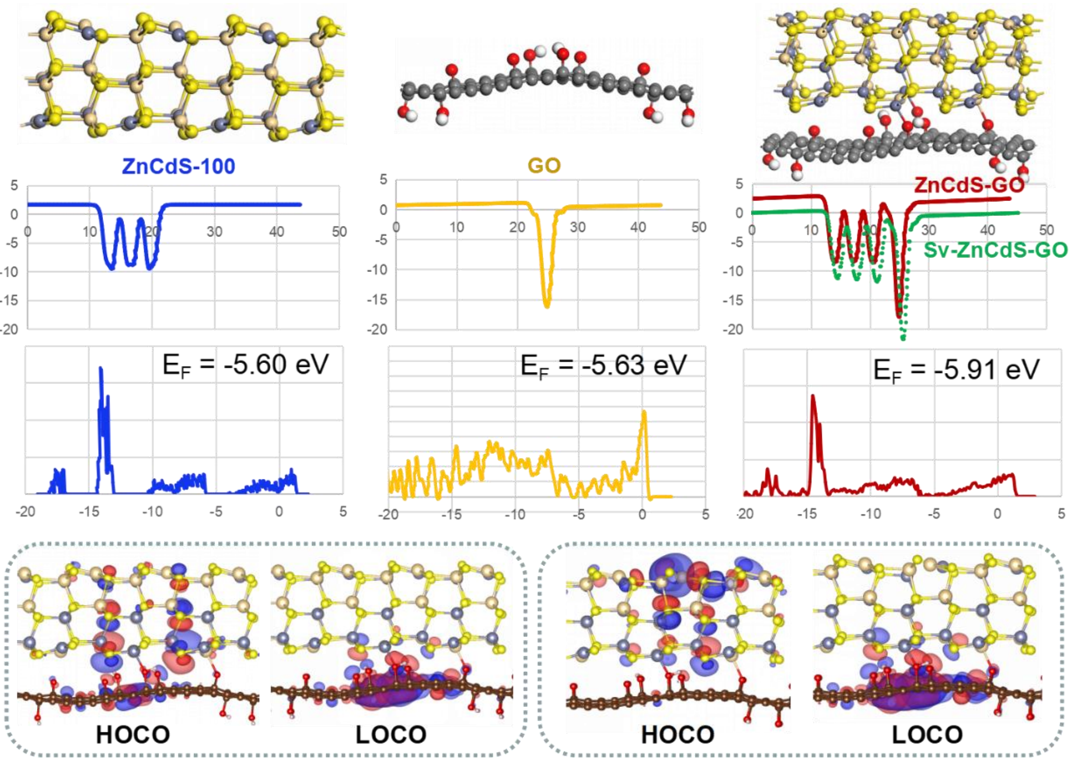

Figure S12. The doping of $V_{S\text{-}2}$ enhanced the local electrostatic potential.

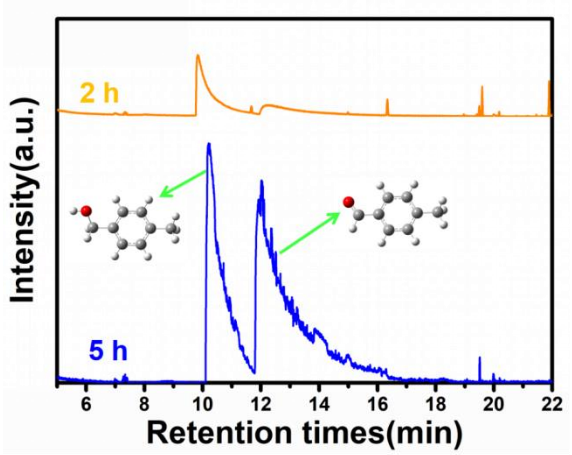

Figure S13. Gas chromatographic of photocatalytic products mixture at 2 h and 5 h with the retention time at 10.19 and 11.92 min respectively.

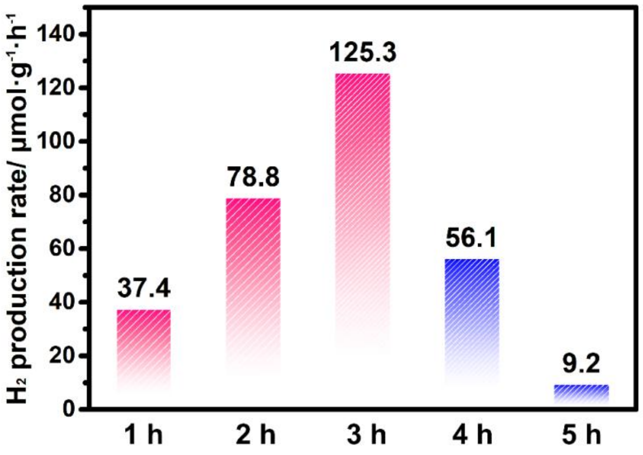

Figure S14. H_2 production rate at different time during cascade reaction.

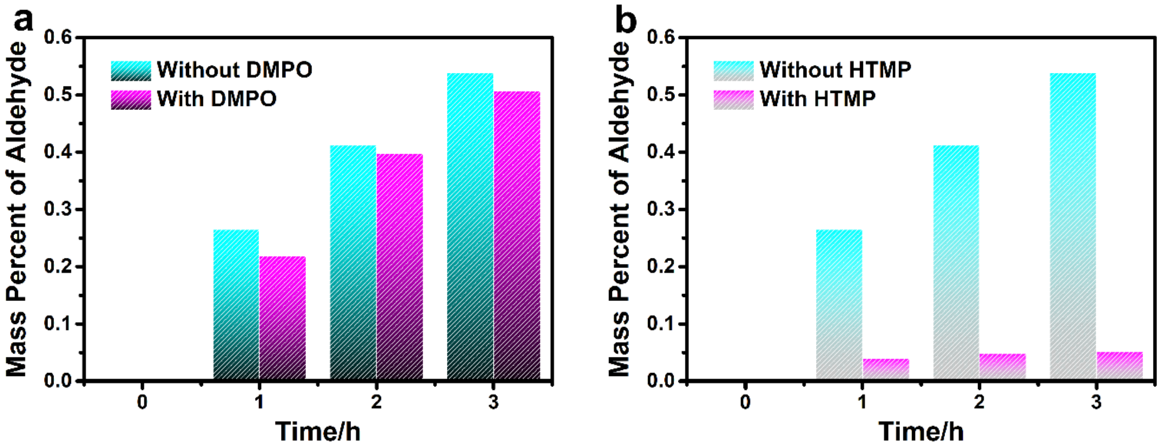

Figure S15. Reactive oxygen species capture experiments of •OH, $•O_2$ with addition of DMPO and $^1O_2$ with addition of HTMP.

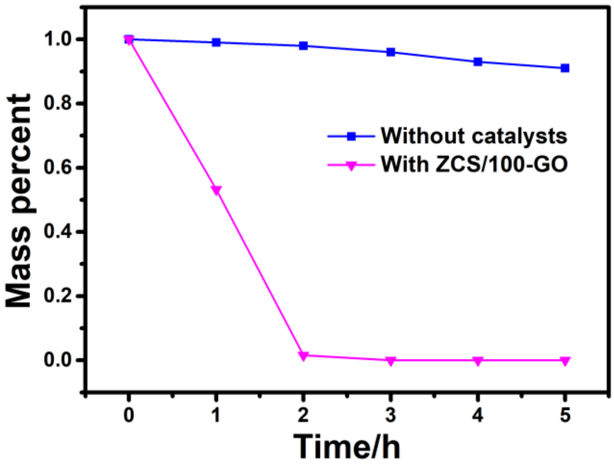

Figure S16. Control experiment of the self-oxidation of p-xylene under light irradiation without photocatalysts.

Table S1 The Bader charge of atoms at different adsorption sites of ZnCdS/GO for oxygen molecule.

Sites \ Atoms	Cd2	Cd8	Zn64	Zn70	C92	C101
ZnCdS-rGO	11.16	11.16	11.16	11.16	3.70	3.70
Cd-O_2	11.15	11.16	11.16	11.16	3.75	3.72
Cd-OOH	11.09	11.16	11.16	11.16	3.73	3.72
GO-O_2	11.16	11.17	11.16	11.16	3.73	3.70
GO-OOH	11.16	11.16	11.17	11.16	3.70	3.69
Zn-O_2	11.17	11.16	11.14	11.16	3.72	3.72
Zn-OOH	11.16	11.17	11.11	11.16	3.74	3.71

Table S2 The Bader charge of atoms at different adsorption sites of V_S-ZnCdS/GO for oxygen molecule

Sites \ Atoms	Cd2	Cd8	Zn64	Zn70	C92	C101	Cd3C-2nd	Zn3C-2nd
V_S-ZnCdS-rGO	11.69	11.17	11.17	11.16	3.75	3.73	11.31	11.22
V_S-Cd3C-O_2	11.63	11.13	11.16	11.17	3.74	3.74	11.29	11.21
V_S-Cd-OOH	11.57	11.10	11.16	11.17	3.72	3.71	11.27	11.22
V_S-GO-O_2	11.67	11.17	11.16	11.16	3.68	3.71	11.31	11.21
V_S-GO-OOH	11.65	11.17	11.17	11.16	3.68	3.69	11.29	11.21
V_S-Zn-O_2	11.68	11.17	11.15	11.16	3.73	3.71	11.31	11.21
V_S-Zn-OOH	11.67	11.17	11.09	11.16	3.75	3.70	11.29	11.22
V_S-O_2	10.97	11.16	11.16	11.16	3.73	3.61	11.04	11.07
V_S-OOH	11.00	11.16	11.11	11.12	3.66	3.74	11.07	11.04

Table S3 The adsorption and desorption energies of O_2 molecule and $\cdot O_2^-$ on (V_S)-$Zn_{0.5}Cd_{0.5}S$/GO

Material	E (eV)	E-mol (eV)	E-slab (eV)	Eads (eV)	Sites
ZnCdS2-GO-Cd-O2	-882.274	-9.857	-872.309	-0.11	Cd3C
ZnCdS2-GO-Zn-O2	-882.259	-9.857	-872.309	-0.09	Zn3C
ZnCdS2-GO-GO-O2	-881.941	-9.857	-872.309	0.22	GO
ZnCdS2-GO-Sv-2-O2	-877.994	-9.857	-865.910	-2.23	Sv
ZnCdS2-GO-Sv-Zn3C-O2	-875.577	-9.857	-865.910	0.19	Zn3C
ZnCdS2-GO-Sv-Cd3C-O2	-876.085	-9.857	-865.910	-0.32	Cd3C
ZnCdS2-GO-Sv-GO-O2	-875.599	-9.857	-865.910	0.17	GO
ZnCdS2-GO-Cd-OOH	-885.973	-13.259	-872.309	-0.40	Cd3C
ZnCdS2-GO-Zn-OOH	-886.557	-13.259	-872.309	-0.99	Zn3C
ZnCdS2-GO-GO-OOH	-885.789	-13.259	-872.309	-0.22	GO
ZnCdS2-GO-Sv-2-OOH	-883.105	-13.259	-865.910	-3.94	Sv
ZnCdS2-GO-Sv-Zn3C-OOH	-880.043	-13.259	-865.910	-0.87	Zn3C
ZnCdS2-GO-Sv-Cd3C-OOH	-880.140	-13.259	-865.910	-0.97	Cd3C
ZnCdS2-GO-Sv-GO-OOH	-878.881	-13.259	-865.910	0.29	GO

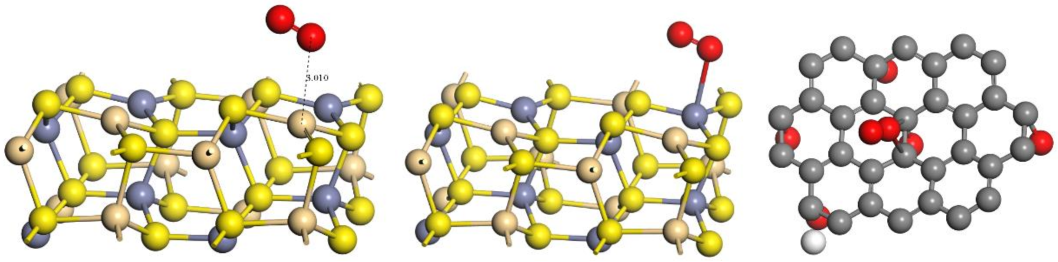

Control group

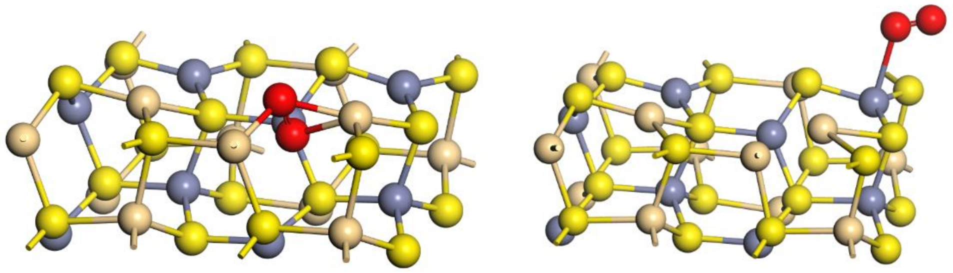

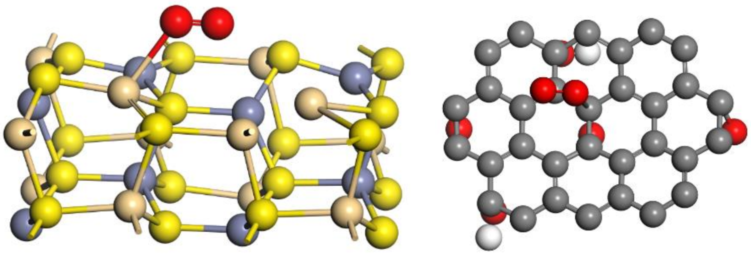

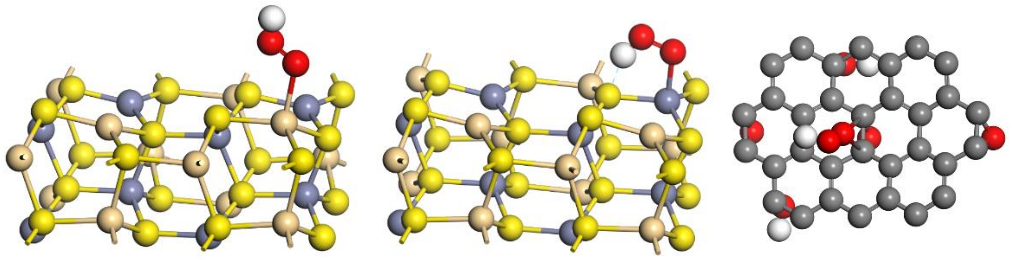

Control group

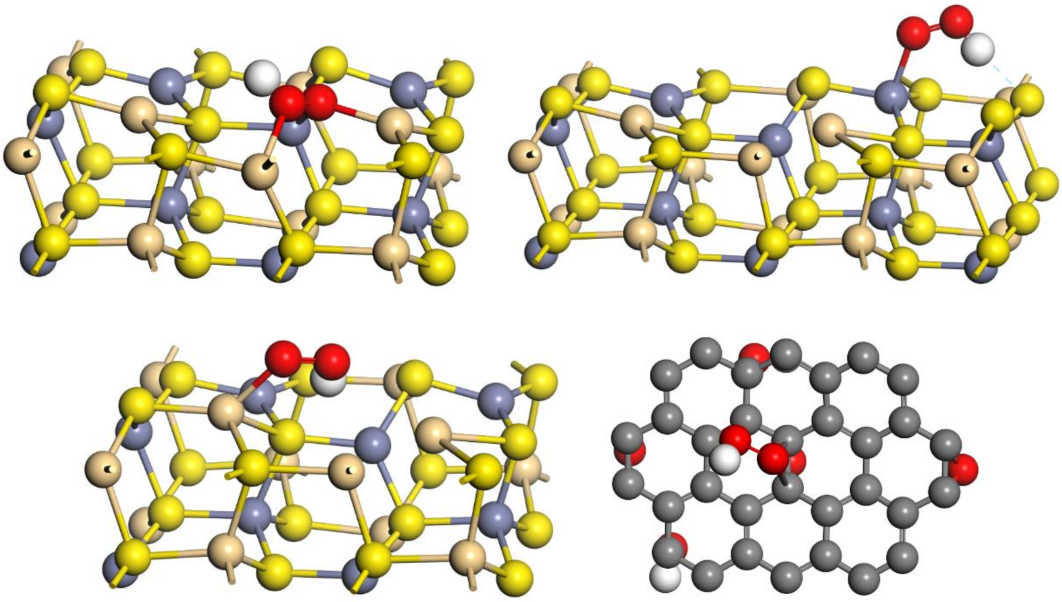

Supplement: Supplementary file 1 — Supporting Information [file ADVS-10-2207250-s001.pdf]
